# Molecularly Imprinted Polymers for the Determination of Cancer Biomarkers

**DOI:** 10.3390/ijms24044105

**Published:** 2023-02-18

**Authors:** Greta Pilvenyte, Vilma Ratautaite, Raimonda Boguzaite, Arunas Ramanavicius, Roman Viter, Simonas Ramanavicius

**Affiliations:** 1 Department of Nanotechnology, State Research Institute Center for Physical Sciences and Technology, Saulėtekio Av. 3, LT-10257 Vilnius, Lithuania; 2 Department of Physical Chemistry, Institute of Chemistry, Faculty of Chemistry and Geosciences, Vilnius University, Naugarduko Str. 24, LT-03225 Vilnius, Lithuania; 3 Institute of Atomic Physics and Spectroscopy, University of Latvia, 19 Raina Blvd., LV-1586 Riga, Latvia; 4 Center for Collective Use of Scientific Equipment, Sumy State University, 31, Sanatornaya St., 40018 Sumy, Ukraine; 5 Department of Electrochemical Material Science, State Research Institute Center for Physical Sciences and Technology, Saulėtekio Av. 3, LT-10257 Vilnius, Lithuania

**Keywords:** molecularly imprinted polymer (MIP), conducting polymer (CP), electrochemical sensor, disease biomarkers, prostate cancer biomarker, breast cancer biomarker, epithelial ovarian cancer biomarker, hepatocellular carcinoma biomarker, small molecule cancer markers, cancer biomarker

## Abstract

Biomarkers can provide critical information about cancer and many other diseases; therefore, developing analytical systems for recognising biomarkers is an essential direction in bioanalytical chemistry. Recently molecularly imprinted polymers (MIPs) have been applied in analytical systems to determine biomarkers. This article aims to an overview of MIPs used for the detection of cancer biomarkers, namely: prostate cancer (PSA), breast cancer (CA15-3, HER-2), epithelial ovarian cancer (CA-125), hepatocellular carcinoma (AFP), and small molecule cancer biomarkers (5-HIAA and neopterin). These cancer biomarkers may be found in tumours, blood, urine, faeces, or other body fluids or tissues. The determination of low concentrations of biomarkers in these complex matrices is technically challenging. The overviewed studies used MIP-based biosensors to assess natural or artificial samples such as blood, serum, plasma, or urine. Molecular imprinting technology and MIP-based sensor creation principles are outlined. Analytical signal determination methods and the nature and chemical structure of the imprinted polymers are discussed. Based on the reviewed biosensors, the results are compared, and the most suitable materials for each biomarker are discussed.

## 1. Introduction

Developing sensors and biosensors is one of the most recent directions of analytical chemistry; therefore, sensor development technologies are evolving rapidly [1]. Most of the challenges in sensors are related to the sensitivity and selectivity of the recognition system [2]. Polymers are frequently used to advance the analytical performance of sensors [3,4,5], particularly for the selectivity towards targeted analytes [3,6,7,8,9]. Some polymer-based structures have unique electrical conductivity [10,11,12,13] and can be applied for the modification of different signal transducers [14,15]. Chemical [16], electrochemical [11], enzymatic [17], and/or microorganism-assisted [18] conducting polymer formation methods are the most frequently used for the formation of unique polymeric structures. For the design of the biological recognition system, various biomaterials (e.g., enzymes [19,20], antigens [21], antibodies [3,17,22], and various receptors [23]) are immobilised within and/or over polymers to increase the selectivity of developed biosensors. However, these materials are not very stable and are mostly very expensive; therefore, there are demands to design cheaper and more stable biorecognition systems suitable for sensor designs. The most attractive way to replace natural biological recognition systems is based on applying molecularly imprinted polymers (MIPs) [3,24]. Electrostatic and hydrophobic interactions between monomers and template molecules play an important role in the specific binding of the template molecules on the MIP [25,26]. The removal of template molecules is a critical step in the preparation of most MIPs [27]. “Gate effect” is used to explain the electrochemical readout of the MIP sensors [28,29,30]. Among various MIP-based structures, electrically conducting polymer-based structures are used [3,31,32,33,34]. The great potential and interest of the population gained the diagnostics from the point-of-care devices, which enable self-health monitoring and management [35]. Meanwhile, Bhakta and Mishra [36] emphasised three main challenges that one can meet during the development of MIPs. These challenges are related to (i) some difficulties occurring during the protein imprinting within the polymer, (ii) some difficulties that occur in the template extraction step, and finally, (iii) the uniformity of the polymer matrix. 

This review article aims to overview the application of MIPs in determining cancer biomarkers and/or diagnosis of some cancerous diseases.

## 2. The General Mechanism and the Preparation Process of MIP-Based Biosensors

**Figure 1:**
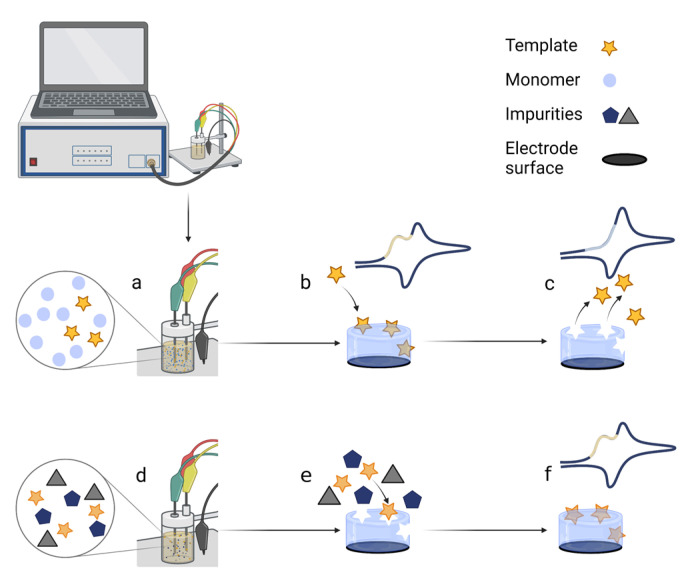
Scheme of the electrochemical polymerisation of the MIP directly on the electrode and the application of the MIP-based electrochemical biosensor: (**a**) polymerisation mixture preparation; (**b**) polymerization of the polymer with template imprints layer on the electrode; (**c**) extraction of the template from the MIP; (**d**) application of the MIP-based sensor; (**e**) competitive interaction of target molecules on the MIP in presence of interferents; (**f**) detection of target molecules. All drawings were designed using Biorender.com, accessed on 26 December 2022.

MIPs are used as artificial receptors to detect target molecules in sensors that act as smart electrochemical output devices. Preparation of the MIP involves several steps (Figure 1): (a) Functional monomers and template molecules are dissolved in an appropriate solvent. (b) A polymerisation process takes place, during which a layer of polymer with template molecules inside is deposited on the surface of the electrode. (c) The template molecules are washed out of the polymer film, leaving imprinted cavities as specific footprints, and the response signal shows no current template peaks. (d) The MIP-based sensor can then be used to detect the target molecules from the sample. (e) Various molecules of a complex sample are exposed on the MIP surface, and only the target molecules enter the imprints. (f) The signal response indicates the detection of the target molecules.

Multi-template [37] methods have also been developed, allowing MIP-based biosensors to detect multiple target analytes in a single complex sample. Various nanomaterials can be used to modify the electrode surface, such as gold nanoparticles [38,39] or nanocomposites containing two or more nanomaterials [37]. As a result, the surface area, conductivity, and, most importantly, sensitivity of the response, increase. Other electrode pretreatment methods, such as the glutaraldehyde-cysteamine layer, which boosts the stability and resilience of the MIP layer [40], self-assembled cysteamine [41], and 8-amino-1-octanethiol [42], are used to make covalent bonds for better analyte binding. Aptamers are used for the enhancement of selectivity and sensitivity [43,44]. The high specificity, selectivity, sensitivity for target molecules, and detection ability of MIP-based electrochemical biosensors open vast possibilities for disease diagnostics. 

## 3. MIP Application for the Detection of Cancer Biomarkers

Proteins and/or some other compounds produced explicitly by cancer cells at larger quantities than those produced by healthy cells have traditionally been used as cancer biomarkers. In some patients with cancer, these proteins may be found in tumours, blood, urine, faeces, or other body fluids or tissues. Tumour biomarkers can reveal information about cancer, such as its aggressiveness, the type of treatment it may respond to, or even whether it is responding to treatment. Most cancer biomarkers are not highly specific, and a positive result does not always imply that the original location may be quickly recognised [45]. MIPs have enabled novel diagnostic techniques for various specific biomarkers of many diseases [46]. In the case of cancer, in addition to the qualitative and quantitative determination of the specific biomarkers of cancer, it is also helpful to determine the biomarkers of the inflammatory process, which according to Brenner et al. [47] belong to classes of inflammation-related biomarkers: cytokines/chemokines, immune-related effectors, acute-phase proteins, reactive oxygen and nitrogen species, prostaglandins and cyclooxygenase-related factors, and mediators (e.g., transcription factors and growth factors). The following are the protein biomarkers of the tumour that are commonly used to diagnose and monitor the treatment of certain cancers (Table 1). New tumour biomarkers frequently become available and may not be included on this list [48].

### 3.1. Biomarker of Prostate Cancer—PSA

A prostate-specific antigen (PSA) is a protein produced by normal and malignant prostate gland cells. The PSA test measures the amount of PSA in the blood. Any PSA value greater than 10.0 ng/mL is considered a positive case, indicating a high risk of prostate cancer. Meanwhile, PSA values ranging from 4.0 to 10.0 ng/mL are a ‘grey area’ and indicate a higher-than-average risk of prostate cancer; thus, more tests may be needed. Lastly, PSA values below 4.0 ng/mL are considered negative, and such PSA values indicate a low risk of prostate cancer [49].

**Figure 2:**
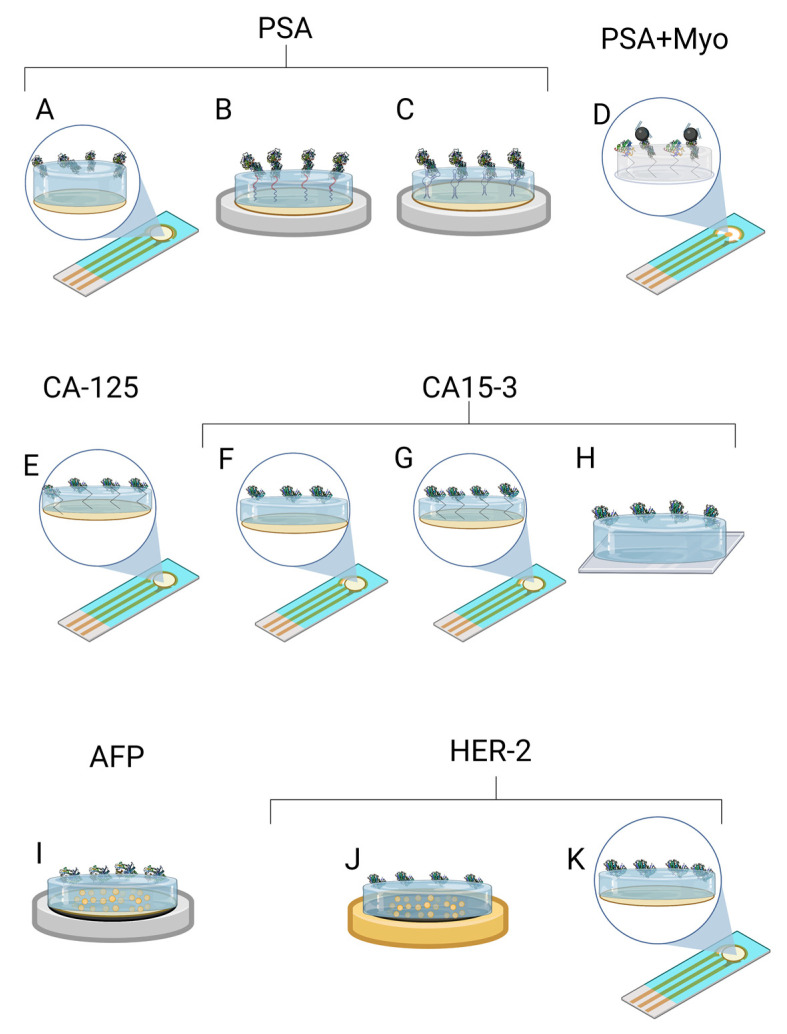
MIP application for detection of cancer biomarkers. All drawings were designed using Biorender.com based on the reviewed articles. (**A**) The Ppy-based MIP with PSA imprints on a gold screen-printed electrode. (**B**) A gold electrode with glutaraldehyde–cysteamine matrix pretreatment coated with polytoluidine blue for analyte PSA entrapment. (**C**) Thiolated aptamer–PSA complex was immobilised on the gold electrode coated with polydopamine for analyte PSA entrapment. (**D**) A screen-printed electrode with self-assembled dithiodipropionic acid di(N-hydroxysuccinimide ester) with immobilised analytes, PSA, and Myo coated with polymethyl acrylate for imprinting. To detect PSA, MIP was exposed to Fe_3_O_4_ nanoparticles decorated with multi-walled carbon nanotubes, graphene oxide, and specific antibodies for PSA. (**E**) A gold screen-printed electrode with self-assembled cysteamine coated with Ppy for analyte CA-125 entrapment. (**F**) A gold screen-printed electrode coated with poly(2-aminophenol) for analyte CA15-3 entrapment. (**G**) The MIP based on the toluidine blue (TB) film was polymerised via glutaraldehyde on self-assembled 8-amino-1-octane thiol-modified AuSPE for analyte CA15-3 entrapment. (**H**) A fluorine-doped tin oxide-glass electrode coated with Ppy for analyte CA15-3 entrapment. (**I**) A glassy carbon electrode with polythionine and gold nanoparticles coated with polydopamine for analyte AFP entrapment. (**J**) Laser-scribed graphene electrode with gold nanoparticles coated with PEDOT for analyte HER-2 entrapment. (**K**) A gold screen-printed electrode coated with polyphenol for analyte HER-2 entrapment. All drawings were designed using Biorender.com, accessed on 26 December 2022.

A novel dual-modality MIP-based biosensor for PSA and myoglobin (Myo) was demonstrated in a study by Karami et al. [37] (Figure 2D). The proposed electrochemical sensor was dedicated to simultaneously detecting two target analytes in serum and urine. For this purpose, a disposable gold screen-printed electrode (SPE) surface was combined with a molecular imprinting technique and an immunoassay approach. The MIP was obtained via free-radical polymerisation of methyl acrylate as a monomer and N, N’-methylenebisacrylamide as a crosslinker in the presence of the nanocomposite, which was made of Fe_3_O_4_ nanoparticles decorated with multi-wallet carbon nanotubes (MWCNT), graphene oxide, and specific antibodies for PSA. LOD of PSA and Myo was found to be 5.4 pg/mL and 0.83 ng/mL, respectively. The selectivity of the proposed sensor was evaluated regarding a relatively long list of interferents. In total, nine interferents were used: bovine serum albumin, cortisol, epidermal growth factor receptor, carcinoembryonic antigen, thrombin antigen, human thrombin, vascular endothelial growth factor, human albumin serum, and neuron-specific enolase. The selectivity test demonstrated that the proposed sensor is highly specific for the target analytes. The accuracy, reproducibility, stability, and regeneration test of the sensor and immunosensor are very promising for everyday analysis. In real samples, recovery of PSA and Myo was favourable.

For PSA detection, Abbasy et al. [40] developed a MIP biosensor for monitoring PSA using a poly(toluidine blue) on a modified AuE. The imprinted poly(toluidine blue) film was electropolymerised on the surface of an electrode in a pre-formed glutaraldehyde-cysteamine (GA-Cys A) matrix (Figure 2B). Due to the cross-linking procedure via the Au–S bonds, the resilience of the MIP against degradation was improved. According to the differential pulse voltammetry method, the biosensor showed a linear response from 0.001–0.06 μg/mL and a LOD of 0.001 μg/mL. This sensor was used to measure PSA in the plasma samples. Sadly, this sensor’s selectivity was tested regarding only two substances: human cancer antigen CEA and human serum albumin. Yazdani et al. [50] developed a more precise sensor by choosing the Py as a functional monomer and was electropolymerised on an AuSPE. Since organic and alkaline solvents could not be used due to the lack of stability of the SPEs, the PSA template was removed overnight with an oxalic acid solution. The LOD of 2.0 pg/mL and a linear range of 0.01–4 ng/mL were obtained (Figure 2A).

Nevertheless, this study did not demonstrate any selectivity tests with interfering substances. Results of the blood serum samples and simple method confirm that the nano biosensor could be successfully used in clinical diagnosis to detect a PSA. Jolly et al. [43] developed a more complex hybrid MIP/aptamer sensor for PSA (Figure 2C). First, a thiolated aptamer–PSA complex was immobilised on the AuE, then the dopamine was electropolymerised, and the PSA template was removed. Polydopamine (PDA) exhibits melanin-like conducting properties and can be used to prepare energy devices such as supercapacitors. PDA can provide numerous ways to provide bioelectronic sensors with excellent biocompatibility, self-repair ability, universal coating capability, and electrical conductivity [51]. The use of aptamers enhances selectivity and specificity. The LOD was 1pg/mL. Such aptamer-based MIPs for PSA were tested in a selectivity test regarding the capacitance changes when the human Kallikrein 2 protein and human serum albumin were used. The authors state that the human Kallikrein 2 protein (or Human glandular Kallikrein 2) is 80% homologous to PSA. The selectivity test demonstrated that the proposed sensor was selective and sensitive to PSA when the interfering substances were used in the samples. Tamboli et al. [44] improved this work with a MIP-based electrochemical sensor using a MOSFET method. The hybrid synthetic receptors were created by immobilising an aptamer–PSA complex on the gold electrode and putting it through multiple cycles of dopamine electropolymerisation. Highly specific cavities were formed, resulting in a very low LOD of 0.1 pg/mL and 1–10 pg/mL in diluted human plasma.

The reviewed articles show that the PDA matrix on the AuE may be the most suitable model for PSA detection as they result in the lowest LOD value [43,44]. The MOSFET method provides precision, making LOD ten times lower than by using differential pulse voltammetry. The biosensor with Ppy is easy to fabricate, but washing takes time, and the resulting LOD is similar to that of the biosensor based on PDA. A dual-modality MIP-based biosensor shows promising results but is challenging to prepare, and antibody stability decreases with increasing temperature. The least sensitive sensor was obtained using a polytoluidine blue. A comparison of the selectivity tests used in the reviewed studies demonstrates that selectivity is the under-evaluated aspect of the electrochemical sensor development. The selectivity tests demonstrate the MIP’s significant precedence over the NIP, but a higher number of interfering substances could often be used in the interfering molecules test. All reviewed studies for PSA detection used AuE or AuSPE.

### 3.2. Biomarkers of Breast Cancer

#### 3.2.1. CA15-3

Cancer antigen 15-3 (CA15-3) is a protein mainly produced by specific breast cancer cells. Some people with breast, lung, pancreatic, ovarian, and prostate cancer or non-cancerous diseases, such as endometriosis, pelvic inflammatory disease, liver disease, or during pregnancy, have higher levels of it in their blood. The increase in CA15-3 is most commonly due to breast cancer with metastases [52] and higher levels (≥30 U/mL values in the blood are considered high) when cancer has spread to the bones, liver, or both [53].

In the study of Ribeiro et al. [42], a disposable biosensor for breast cancer biomarker CA15-3 recognition via molecular imprinting using polytoluidine blue as a conducting film was described (Figure 2G). The polytoluidine blue film was electropolymerised via glutaraldehyde on a preformed SAM on an AuSPE. This structure gave greater stability to the adhesion of the film on the electrode surface. The biosensor results showed a linear response from 0.10 U/mL to 100 U/mL and a LOD below 0.10 U/mL. Given that CA15-3 is a high molecular weight glycoprotein (300–450 kDa), the selectivity assay of diluted artificial serum containing small molecules ranging from 58 to 180 Da and one larger molecule, such as bovine serum albumin (66 kDa), does not appear to be sufficient to compare precise selectivity. However, with further studies, this new biosensor appears to have the potential for point-of-care use, as it has a wide concentration range that allows direct analysis of samples above or well below the cut-off value of 30 U/mL. Santos et al. [54] also conducted a polymer to develop a sensor for CA15-3. Pyrrole was electropolymerised on a fluorine-doped tin oxide conductive glass. Ethanol was chosen for easy template removal. The biosensor resulted in a LOD of 1.07 U/mL, with a linear response of 1.44–13.2 U/mL. This sensor’s selectivity was evaluated using only two molecules: carcinoembryonic antigen and interleukin 6. Pacheco et al. [55] developed a MIP-based sensor to detect CA15-3 with a non-conducting poly(2-aminophenol) film on the surface of an AuE that resulted in a LOD of 1.5 U/mL (Figure 2F). Another breast cancer biomarker, the human epidermal growth factor receptor 2 extracellular domain, and cystatin C, a kidney function biomarker, were chosen to evaluate selectivity, and the results indicated that the human epidermal growth factor receptor 2 extracellular domain may influence the analysis. This MIP sensor provides easy-to-prepare, low-cost monitoring but with lower sensitivity than the sensors used in the previously mentioned studies. 

AuSPE and FTO glass were included in the reviewed studies for CA15-3 detection. AuSPE with a polytoluidine blue matrix [42] resulted in the lowest LOD, following fluorine-doped tin oxide glass with a Ppy matrix [54], resulting in a similar LOD. The biosensor with a non-conducting polymer matrix poly(*o*-aminophenol) [55] showed the least sensitive detection compared to previous MIP sensors with polytoluidine blue and Ppy. In conclusion, based on the LOD, these electrochemical MIP biosensors demonstrate that molecular imprinting is suitable for detecting breast cancer CA15-3 biomarkers.

#### 3.2.2. HER-2

Human epidermal growth factor receptor 2 (HER-2) is a significant biomarker in invasive breast cancer, according to the 2007 American Society of Clinical Oncology guidelines. Clinical HER-2 testing is performed using immunohistochemistry, ELISA analysis of serum or tumour cytosol, and the Western blot test for HER-2 protein overexpression. Immunohistochemistry is the most frequently used test to evaluate the HER-2 status in breast cancer and screening [56], though there are issues with the accuracy and consistency of immunohistochemistry results acquired from different laboratories. The ELISA approach is expensive and lacks clinical evidence for prognostic usefulness or comparability to other tests [57]. The cut-off value is 15.0 ng/mL for the serum-HER-2 concentration [58].

Lahcen et al. [38] developed a point-of-care nanostructured sensor for the cancer biomarker HER-2 (Figure 2J). The biosensing platform was fabricated of laser-scribed graphene (LSG) electrodes modified with nanostructured gold and MIP. LSG has high electrical and thermal conductivity, excellent mechanical stability, and a large specific surface area. The gold nanostructures enhance the surface area and promote protein adsorption, and MIP enhances selectivity and sensitivity. The functional monomer for conductive MIP was chosen from Py, aminothiophenol, and 3,4-ethylenedioxythiophene. First, the pyrrole was polymerised on LSG–gold nanostructures, and the results showed increased charge transfer resistance. The plausible reason for this was the too-thick Ppy layer. Next, polyaminothiophenol showed better interaction with the gold nanostructures layer due to thiol groups, but the peak current response was significantly hindered. Finally, the poly(3,4-ethylenedioxythiophene) polymer showed the best results among the three polymers on LSG–gold nanostructures. The achieved LOD of 0.43 ng/mL is clinically relevant for monitoring the breast cancer biomarker HER-2. The selectivity of the biosensor was tested in the presence of seven other molecules such as cardiac troponin-I, cardiac troponin-T, cardiac troponin-C, glucose, dopamine, myoglobin, and cholesterol. The results showed suitable selectivity for HER-2. Pacheco et al. [59] developed a sensor for breast cancer biomarker (HER-2-ECD) detection using a molecularly imprinted electrochemical sensor with non-conducting polymer polyphenol (Figure 2K). The LOD was 1.6 ng/mL, higher than the previous conducting MIP sensor. The sensor’s response to two interfering proteins was tested for the selectivity evaluation: CA 15-3 and cystatin C. The results showed that CA 15-3 may interfere with the analysis.

Both methods used gold, one combining AuSPE with a polyphenol matrix [59], the other using LSG with gold nanostructures and a poly(3,4-ethylenedioxythiophene) [38]. The results showed that the sensor with poly(3,4-ethylenedioxythiophene) had a lower LOD of HER-2 than the non-conducting polyphenol.

### 3.3. Biomarker of Epithelial Ovarian Cancer—CA-125

Cancer antigen 125 (CA-125) is a standard tumour biomarker used to diagnose epithelial ovarian cancer and monitor women who have been diagnosed with epithelial ovarian cancer during or after therapy. Because of its stability and widespread availability, CA-125 is still regarded as the best available biomarker for epithelial ovarian cancer. According to the clinical guidelines for ovarian cancer published by the National Institute for Health and Care Excellence, the most frequently quoted reference range for CA-125 in the diagnosis of epithelial ovarian cancer is 0–35 IU/mL [60]. The label-free amperometric immunosensor [61] is one of many approaches used with immunoassays. Measurements of CA-125 can be made using immunoradiometric assays, electrochemical immunoassays, ELISA, electrochemical immunosensors, electrochemiluminescence immunosensors on paper, quartz crystal microbalance, surface plasmon resonance, and plasmon resonance scattering. While sensitive, immunoradiometric tests, enzyme immunoassays, and ELISA are also complex, time-consuming, expensive, and imprecise, and toxically reagent-intensive. Immobilisation of immunoreagent onto the electrode surface is another issue with the electrochemical enzyme immunoassay [62].

Rebelo et al. [41] designed a MIP to determine CA-125 via the electropolymerisation of pyrrole on an AuSPE. For quantification, the square wave voltammetry and surface plasmon resonance were used. Results showed that biosensors based on electrochemical transduction presented a better performance with a lower LOD of 0.01 U/mL and a more extensive linear range between 0.01 and 500 U/mL. The sensor’s analytical performance was tested in an artificial serum medium to compare it to the results obtained in the buffer solution. Exposing the MIP sensor to artificial serum increased sensitivity, LOD (0.1 U/mL), and the linear concentration range was reduced, resulting in 0.1–500 U/mL. The quantification of CA-125 in serum is achievable even with a reduced working range because its threshold value is 35 U/mL. Furthermore, selectivity was demonstrated using interferences: CA 15-3 and artificial serum containing NaCl, NaHCO_3_, and bovine serum albumin. This biosensor provides accurate findings for the measurement of CA-125 based on the LOD (0.01 U/mL). For the determination of CA-125, several other detection approaches covering molecular imprinting were described, including a non-conducting polymer MIP sensor using polyphenol [63], resulting in a higher LOD (of 0.5 U/mL) than a reviewed article with a conducting polymer (LOD 0.01 U/mL) [41], magnetic MIP sensor (for CA-125 and CA15-3) [64], and the synthetic receptor built using the molecular imprinting approach [62]. 

### 3.4. Biomarker of Hepatocellular Carcinoma—AFP

Despite the several biomarkers proposed for hepatocellular carcinoma, AFP is still the most widely used criterion. In addition, patients with a bile duct, gastric, lung, certain ovarian and teratocarcinoma, or embryonal carcinoma of the testis carcinoma also have higher serum amounts [52]. 

**Figure 3:**
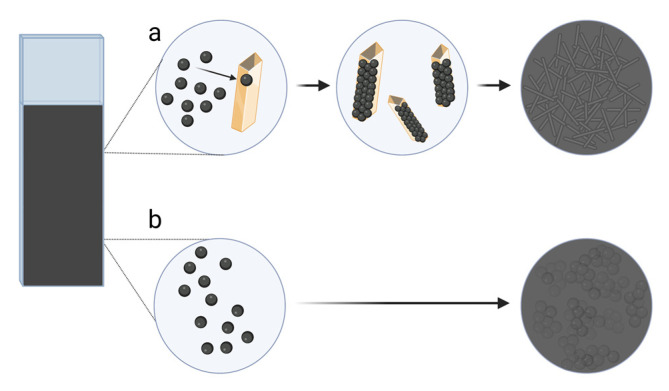
The polymerisation of pyrrole (**a**) in the presence of methyl orange, (**b**) without dyes. (**a**) Electropolymerisation of pyrrole in the presence of methyl orange. Methyl orange creates a rectangular template for attaching pyrrole monomers. This results in the formation of polypyrrole rectangular nanotubes with more surface area. (**b**) Electropolymerisation of pyrrole without dyes, resulting in a polypyrrole with rounder edges and less surface area. All drawings were designed using Biorender.com, accessed on 26 December 2022.

AFP is a 70-kDa glycoprotein that transports bilirubin, fatty acids, retinoids, steroids, heavy metals, phytoestrogens, flavonoids, and perhaps medicines. Except for pregnant women, serum AFP levels rapidly decline after birth, leaving only negligible amounts (5 ng/mL) in healthy individuals [65]. According to the American Board of Internal Medicine, the reference range in serum is ˂10 ng/mL [66]. The 400 ng/mL AFP threshold in serum is more accurate in detecting hepatocellular carcinoma than the 200 ng/mL criterion [67]. Lai et al. [39] introduced a modified electrochemical MIP immunosensor for the detection of AFP (Figure 2I). MIP was prepared via electropolymerisation of the dopamine using AFP templates on a conducting polymer polythionine film with a fast charge transfer capacity to detect the AFP target molecules. Modifying GCE with gold nanoparticles and polythionine provided high conductivity, stability, and adequate biocompatibility, resulting in a low LOD of 0.8138 pg/mL. Good sensor recoveries were obtained using spiked human serum samples, although the selectivity of the biosensor was not investigated. With further improvements, the sensor may be suitable for monitoring AFP in patients. A dual MIP-based biosensor was developed for the AFP and carcinoembryonic antigen (CEA) [68]. For template imprinting, electropolymerisation of Py on the FTO electrode was performed in the presence of methyl orange, the AFP, and CEA antigens. Pyrrole monomers polymerise around the methyl orange template due to electrostatic interactions and hydrogen bonding. Methyl orange is vital in producing molecularly imprinted Ppy because it promotes the formation of Ppy rectangular nanotubes (Figure 3). Ppy conductivity increases with the increasing specific surface area, regardless of morphology [69], resulting in higher sensor sensitivity. CEA and AFP had linear ranges of 5–10^4^ and 10–10^4^ pg/mL and detection limits of 1.6 and 3.3 pg/mL, respectively. The biosensor provided accurate results for AFP and CEA determination in serum samples, as well as high sensitivity and stability over five weeks period. The selectivity of the developed sensor was evaluated against various interferences, PSA, HE4, CA19-9, CA125, and CA15-3, and showed excellent results [68].

A summary of the overviewed studies is given in Table 2 and includes information about polymers used in the design of MIP, type of electrode, method of analysis for the detection of biomarkers, and analytical parameters such as linear range and LOD.

### 3.5. Detection of Small Molecule Cancer Biomarkers: 5-HIAA and Neopterin

Cancer can be suspected not only by detecting proteins but also by small-weight molecules. For example, 5-Hydroxyindole acetic acid (5-HIAA) is a serotonin metabolite [70]. Therefore, 5-HIAA is used to evaluate serotonin levels from urine samples. One of the reasons for elevated levels of 5-HIAA is a carcinoid tumour. Typical results are 2–9 mg of 5-HIAA over 24 h [66]. Generally, women have lower levels than men.

Moncer et al. [71] reported the development of an electrochemical biosensor on GCE for 5-HIAA detection based on molecularly imprinted Ppy. Choosing a suitable solvent for washing is one of the most crucial steps in preparing MIPs. It creates complementary imprinting cavities for selective rebinding by washing the templates and preserving the same three-dimensional polymer network structure. Using NaOH (0.1 to 0.2 M) as a washing solution results in a partial or even total detachment of the Ppy film from the GCE surface. This issue could be caused by gaseous species formation that destroys the adhesion of the film to the electrode surface [72]. Therefore, the relatively lower (0.05 M) concentration of NaOH was chosen to give the most efficient removal of 5-HIAA and ensure a good nanofilm adhesion to the electrode. The selectivity was tested with interfering compounds: tryptophan, tyrosine, serotonin, indole, indole-3-acetic acid, and l(+)-ascorbic acid. In addition, the interference of paracetamol, amoxicillin, ibuprofen, 17β-estradiol, and caffeine was also investigated due to their ability to distort normal 5-HIAA levels. Finally, a MIP sensor was applied to detect 5-HIAA in plasma, serum, and urine samples with a LOD of 15 pM/L. The results showed that this nano-sensor is a promising tool for the quick screening of 5-HIAA in urine without laborious pretreatment, where its normal levels in urine are found to be 4–9 mg/mL/24 h. This is owing to the characteristics of MIPs that can quickly screen the sample, selectively identify the template, and quantify it with high sensitivity.

Neopterin is another cancer biomarker with a small molecular weight. In addition, it is known as 1′,2′,3′-D-erythro-trihydroxypropylpterin. This is a type of unconjugated pteridine that is found in live cells as a metabolic product [73]. Increased neopterin concentrations are also significant in the progression of other diseases such as virus infections such as COVID-19, as well as in transplant rejection, autoimmune disorders, cardiovascular diseases, neurodegenerative diseases, and cancer. Because of its sensitivity to light, limited solubility, and low concentration in biological fluids, the analysis of neopterin is complicated [73,74]. In serum, the concentration values of neopterin in healthy individuals are up to 10 nM, while greater than 10 nM can be considered elevated [75,76]. A conducting polymer mix of bis–bithiophene derivatised with cytosine and bithiophene derivatised with boronic acid on a Pt electrode was used in a design of a molecularly imprinted sensor to detect neopterin [77]. The sensor’s sensitivity to neopterin was over three-and-a-half times that of pterin, nearly seventy times that of 6-biopterin, and nearly seventeen times that of creatinine. The sensor also did not respond to glucose or xanthine. The sensitivity of chemosensors to neopterin in real samples was similar to that of neopterin in aqueous solutions. The LOD of the sensor was 22 µM.

## 4. Conclusions

The overviewed studies have shown that MIPs can be successfully applied in developing biosensors for cancer biomarkers. To conclude, this review article focuses on the newest research about electrochemical MIP-based biosensors for detecting protein-based and small molecular weight biomarkers for prostate, breast, epithelial ovarian, and hepatocellular cancers. MIP-based biosensors with conducting polymers gave better LODs for PSA, CA15-3, HER-2, CA-125, and neopterin biomarkers determination than non-conducting polymers.

In conclusion, MIP-based electrochemical biosensors were used to detect various cancer biomarkers such as proteins (PSA, Myo, CA15-3, HER-2, CA-125) or small molecules (5-HIAA, neopterin). Therefore, it could be an alternative to expensive and time-consuming laboratory tests. In the reviewed articles, gold was mainly chosen for electrodes. Other types of electrodes, such as GCE and LSG electrodes, were combined with gold nanoparticles. SPE became a common choice recently due to its convenient clinical use. Since the determination of multiple biomarkers is usually required for cancer detection, it would be advantageous to develop biosensors capable of simultaneously detecting several biomarkers. Overall, these biosensors offer a simple, sensitive, and low-cost analysis required for the early diagnosis of cancer in comparison with routine laboratory tests.

In addition to the current pandemic, there is an urgent need for new tools that allow rapid diagnosis, especially in the early stages of cancer, which are increasing worldwide. Many new technical developments of MIP-based biosensors are currently under intense study to help achieve this goal at the multidisciplinary interface of chemistry, biology, and material science. As discussed in this review, MIP-based biosensors have many diagnostic possibilities for detecting various cancer biomarkers, such as proteins (PSA, Myo, CA15-3, HER-2, CA-125) or small-weight molecules (5-HIAA, neopterin) due to their robustness, sensitivity, and inexpensive analysis.

## Figures and Tables

**Table 1:** The summary of commonly used protein biomarkers for tumour diagnosis and monitoring.

Biomarker	Analysed Matrix	Appliance
Breast cancer		
CA15-3	Blood	To assess whether treatment is working or if cancer has recurred.
*HER-2/neu* gene amplification or protein overexpression	Tumour	To help determine treatment.
CA 27.29	Blood	To detect metastasis or recurrence.
Estrogen receptor (ER)/progesterone receptor (PR)	Tumour	To help determine treatment.
Urokinase plasminogen activator (uPA) and plasminogen activator inhibitor (PAI-1)	Tumour	To determine the aggressiveness of cancer and guide treatment.
Ovarian cancer		
CA-125	Blood	To help in diagnosis, assessment of response to treatment, and evaluation of recurrence.
HE4	Blood	To plan cancer treatment, assess disease progression, and monitor for recurrence.
*HER-2/neu* gene amplification or protein overexpression	Tumour	To help determine treatment.
5-Protein signature (OVA1)	Blood	To pre-operatively assess pelvic mass for suspected ovarian cancer.
Prostate cancer		
Prostatic Acid Phosphatase (PAP)	Blood	To help in diagnosing poorly differentiated carcinomas.
Prostate-specific antigen (PSA)	Blood	To help in diagnosis, to assess response to treatment, and to look for recurrence.
Liver/hepatocellular cancer		
α-fetoprotein (AFP)	Blood	To help diagnose liver cancer and follow response to treatment; to assess stage, prognosis, and response to treatment of germ cell tumours.
Programmed death ligand 1 (PD-L1)	Tumour	To help determine treatment.
Des-gamma-carboxy prothrombin (DCP)	Blood	To monitor the effectiveness of treatment and to detect recurrence.

**Table 2:** The summary of MIP application for detection of cancer biomarkers.

Polymer	Electrode	Detection Method	Linear Range	LOD	Ref.
Myo					
Poly(N, N’-methylenebisacrylamide-acrylamide)	AuSPE	EIS	1–20,000 ng/mL	0.83 ng/mL	[37]
PSA					
Poly(N, N’-methylenebisacrylamide-acrylamide)	AuSPE	EIS	0.01–100 ng/mL	5.4 pg/mL	[37]
Ppy	AuSPE	DPV	0.01–4 ng/mL	2.0 pg/mL	[50]
Poly(toluidine blue)	AuE	DPV	1–60 μg/L	1 μg/L	[40]
PDA	AuE	DPV	0.100–100 ng/mL	1 pg/mL	[43]
PDA	AuE	MOSFET	0.1 pg/mL–1 ng/mL	0.1 pg/mL	[44]
CA15-3					
Poly(*O*-aminophenol)	AuSPE	DPV, EIS	5–50 U/mL	1.5 U/mL	[55]
Poly(toluidine blue)	AuSPE	DPV	0.10–100 U/mL	0.10 U/mL	[42]
Ppy	FTO-glass	EMF	1.44 to 13.2 U/mL	1.072 U/mL	[54]
CA-125					
Ppy	AuSPE	SWV	0.01–500 U/mL	0.01 U/mL	[41]
AFP					
PDA	GCE	DPV	0.001–800 ng/mL	0.8138 pg/mL	[39]
Ppy	FTO	CV, EIS	10–10^4^ pg/mL	3.3 pg/mL	[68]
HER-2					
Poly(3,4-ethylenedioxythiophene)	LSG	SWV, EIS	-	0.43 ng/mL	[38]
Polyphenol	AuSPE	EIS, DPV	10–70 ng/mL	1.6 ng/mL	[59]

Ppy—polypyrrole; PDA—polydopamine; AuSPE—gold screen-printed electrode; AuE—gold electrode; FTO—Fluorine-doped Tin Oxide; GCE—glassy carbon electrode; EIS—electrochemical impedance spectroscopy; MOSFET—metal–oxide–semiconductor field-effect transistor; DPV—differential pulse voltammetry; EMF—electromotive force measures; SWV—square wave voltammetry.

## Data Availability

Data sharing is not applicable to this article.
